# Novel perspectives on plant behavior

**DOI:** 10.1080/15592324.2024.2419673

**Published:** 2024-11-04

**Authors:** E. Van Volkenburgh, ED. Brenner

**Affiliations:** Department of Biology, University of Washington, Seattle, WA, USA; Department of Biology, Pace University, New York, NY, USA

## Research into plant behavior gains momentum

Research is rapidly advancing in the emerging areas of plant behavior, unveiling novel frontiers in signaling, communication, and cognition. Recent breakthroughs are illuminating the intricate and dynamic nature of plant interactions. In June 2023, members and friends of the Society for Plant Signaling and Behavior convened in Seattle at the University of Washington, greeting each other after a long Covid-imposed isolation and attracting new participants to the field. The symposium was vibrant and refreshing. In part, it was funded by the US National Science Foundation to support attendance and active participation by emerging women scientists from groups underrepresented in science. Symposium participants and others interested were subsequently invited to contribute to this special issue of *Plant Signaling and Behavior*. The articles presented here range from a critical review of plant neurobiology to studies of inter-plant communication and the intricate interplay among and between plants, pathogens, and herbivores. Importantly, two articles address facets of plant-specific intelligence: cognition that propels complex plant behavior and complex behaviors demonstrating learning and agency.

## Origin of the society of plant signaling and behavior

**Figure 1.**
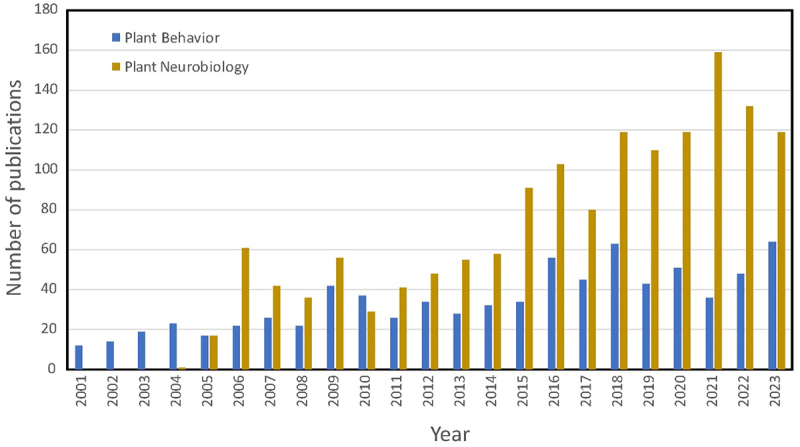
Total number of articles published per year in the scientific literature containing the terms ‘plant neurobiology’ or ‘plant behavior.‘ a search was conducted in Google Scholar with the term ‘plant behavior’ or ‘plant behaviour’ and a second separate search was conducted with the term ‘plant neurobiology’ appearing anywhere in the text. Articles positive for these terms were then filtered by individually examining each article and omitting those unrelated to biological plants, such as power plants.

The formation of the Society coincided with its first international symposium in Florence (2005), and subsequent publication of a controversial article in *Trends in Plant Science*, “Plant Neurobiology, an Integrated View of Plant Biology”^1^. Originally the group was named the Society for Plant Neurobiology to draw attention to the complex information-processing underlying plant behavior. With persistent criticism primarily from a subgroup of mainstream plant biologists, the Society changed its name to reflect the broader scope of plant adaptive responsiveness and behavior. Nevertheless, colloquially and informally, some still appreciate and believe in the appropriateness of utilizing concepts of neurobiology to test hypotheses about plant abilities to perceive, process and integrate information, and how this affects a variety of plant processes including behavior. Reference to ‘plant neurobiology’ has persisted with a renegade flare as the concept continues to be argued back-and-forth.^2^ The term continues to be used in the scientific literature amidst high-quality research ranging from wet – lab work to philosophical treatises.^3,4^ Since 2006 there has been a steady uptick in the number of journal articles addressing the concept of plant neurobiology.* Likewise, there has been a slower yet steady increase in articles explicitly using the term ‘plant behavior’ (Figure 1). From a practical standpoint, the use of the concept ‘plant neurobiology’ continues to provoke interest in plant behavior, inspire research, and most importantly inspire new ways of thinking about plant function and adaptation.^5^

*The first use of the term ‘plant neurobiology’ in the literature in this context was in 2004, where it was presented within quotations, more as a concept than as a field of research.^6^

## Novel perspectives on plant behavior

Whether described as plant neurobiology or plant behavior, the society and its journal, Plant Signaling and Behavior, are designed to develop an understanding of plant function using a holistic approach. The articles in this collection highlight progress being made at the molecular, developmental, ecological, physiological, and philosophical levels, including a perspective on the controversy over ‘plant neurobiology’ as a term to describe the field,^2^ and an overview of possible future directions for research in plant behavior.^7^

Lauren Erland (University of the Fraser Valley) reviews the role of indoleamines, in particular, melatonin and serotonin.^8^ She showcases the diversity and breadth of the research community involved. Indolamines are a class of plant hormones involved in plant responses to environmental stress and communication among plants and animals. In just the past 5 years improvements of quantification methods have allowed focus on interactions among indoleamine and other hormone signaling pathways. Searching for the elusive phytoserotonin receptor promises to jump this field forward. Understanding indoleamine pathways and interactions with other signaling pathways will also be useful for determining the applicability of these metabolites as tools in new agricultural practices.

Bianca Bonato, Valentina Simonetti, Silvia Guerra, and Umberto Castiello (University of Padova) present new information^9^ about an early-documented and classic plant behavior, the^10^ twining of tendrils. They show that plants can discriminate between an artificial and biological support, adjusting their circumnutation patterns on approach to the support. The mechanisms of perception of the supports by the tendrils is speculated by the authors to be visual for the artificial support or a combination of visual and ‘chemical’ (detection of volatile compounds) in the case of the biological support. In the latter case, integration of these two sensory systems would improve accuracy for locating and grasping the support. This model system could provide opportunities for exploring how signaling systems mesh.

Andre Kessler and Michael Mueller (Cornell University) focus on plant mechanisms for priming defense responses to herbivory as a case study in the application of concepts of plant intelligence.^11^ To this end, they address the nature and meaning of ‘intelligence’ as applied to plants. The wealth of sensory and biochemical information available in this field permits at least the beginnings of a working framework for how intelligence may be uniquely constructed in plants.

Peter Minorsky (Mercy University) clarifies the field of plant neurobiology or plant behavioral biology as it could be more justly named.^2^ He identifies the marginalized ‘triad disciplines’ of plant ethology, whole plant electrophysiology, and plant comparative psychology. With eight arguments, he refutes opinions that research in plant neurobiology and behavior is nothing new or that it sits well within the field of plant physiology. With this treatise, it can be hoped that the confusion over the use of ‘plant neurobiology’ is alleviated. Further, the complexity of the field, with all its conceptual layers, becomes exciting when Minorsky describes how plants are capable of executing many of the functional pathways that inspire research into humans.

Michael Marder and Andre Geremia Parise (University of the Basque Country and University of Reading) bring forward the concept of cognition in plants, elucidating how the indeterminate growth pattern of plants bolsters the concept of extending cognition.^4^ By considering specific plant functions including adaptive phenotypic plasticity accomplished by growth, movement, volatile emissions, and root exudation, these authors contribute to the field of post-cognitivism by broadening the theory of extended cognition.

Ariel Novoplansky, Gustavo Maia Souza, Eric D. Brenner, Satish Bhatla, and Elizabeth Van Volkenburgh (Ben Gurion University of the Negev, University of Pelotas, Pace University, University of Delhi, University of Washington) capture the status of research in the field of plant behavior and point out possible future directions.^7^ Earlier emphases on plant movements, molecular and physiological mechanisms including sensory biochemistry (hormonal and other chemical signaling, electrical signaling by cells and through organs, and volatile emissions for communication) are giving way to a higher order of information integration. They make the argument that plants have agency and can learn and remember. Plants call on memory for actions, not only in the increasingly understood mechanisms of priming. That plant behavior relies on complex perception and integration of multiple signals and cues, learning, memory, and dynamic decision-making support an understanding of plant agency. This framework opens possibilities for linking previously well-defined areas of research with new directions utilizing modern methods of data collection and analysis. The case is made that plants are complex knowledgeable beings, suggesting that information about plants’ function and behavior will generate novel developments in engineering and agriculture.

Overall, research into plant behavioral biology has become complex, requiring exposure, interactions, and cross fertilization among scientists and philosophers across multiple disciplines. Not only is it necessary to secure funding to discover as yet unknown forms of plant behavior, the challenge arises in developing research teams sufficiently trained to integrate previously isolated endeavors; such as biochemistry, community ecology, and cognitive studies. As the attraction of students and funding to this field accelerates, the rapid inclusion of modern methods and integrated perspectives will amplify the usefulness of research and knowledge about plant behavior.
